# A plug‐and‐play, lightweight, single‐axis gradient insert design for increasing spatiotemporal resolution in echo planar imaging‐based brain imaging

**DOI:** 10.1002/nbm.4499

**Published:** 2021-02-22

**Authors:** Edwin Versteeg, Tijl A. van der Velden, Carel C. van Leeuwen, Martino Borgo, Erik R. Huijing, Arjan D. Hendriks, Jeroen Hendrikse, Dennis W. J. Klomp, Jeroen C. W. Siero

**Affiliations:** ^1^ Department of Radiology University Medical Center Utrecht Utrecht the Netherlands; ^2^ Futura Composites BV Heerhugowaard the Netherlands; ^3^ Spinoza Center for Neuroimaging Amsterdam the Netherlands

**Keywords:** EPI, gradient coil, insert, magnetic resonance imaging, plug‐and‐play

## Abstract

The goal of this study was to introduce and evaluate the performance of a lightweight, high‐performance, single‐axis (z‐axis) gradient insert design primarily intended for high‐resolution functional magnetic resonance imaging, and aimed at providing both ease of use and a boost in spatiotemporal resolution. The optimal winding positions of the coil were obtained using a genetic algorithm with a cost function that balanced gradient performance (minimum 0.30 mT/m/A) and field linearity (≥16 cm linear region). These parameters were verified using field distribution measurements by B_0_‐mapping. The correction of geometrical distortions was performed using theoretical field distribution of the coil. Simulations and measurements were performed to investigate the echo planar imaging echo‐spacing reduction due to the improved gradient performance. The resulting coil featured a 16‐cm linear region, a weight of 45 kg, an installation time of 15 min, and a maximum gradient strength and slew rate of 200 mT/m and 1300 T/m/s, respectively, when paired with a commercially available gradient amplifier (940 V/630 A). The field distribution measurements matched the theoretically expected field. By utilizing the theoretical field distribution, geometrical distortions were corrected to within 6% of the whole‐body gradient reference image in the target region. Compared with a whole‐body gradient set, a maximum reduction in echo‐spacing of a factor of 2.3 was found, translating to a 344 μs echo‐spacing, for a field of view of 192 mm, a receiver bandwidth of 920 kHz and a gradient amplitude of 112 mT/m. We present a lightweight, single‐axis gradient insert design that can provide high gradient performance and an increase in spatiotemporal resolution with correctable geometrical distortions while also offering a short installation time of less than 15 min and minimal system modifications.

## Graphical abstract

A single‐axis gradient insert, designed to be plug‐and‐play while providing a significant boost in gradient performance (gradient strength = 200 mT/m and slew rate = 1300 T/m/s), was presented. This coil was characterized in terms of linearity, image distortions and reduction in echo‐spacing for echo planar imaging. Distortions were found to be correctable and echo‐spacing could be reduced by a factor of 2.3 compared with a whole‐body gradient set (40 mT/m and 200 T/m/s).

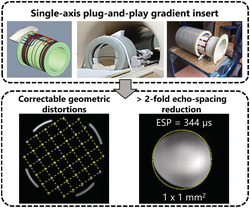

**ABBREVIATIONS USED**
EPI: echo planar imaging
ESP: echo‐spacing
ETL: echo train length
fMRI: functional magnetic resonance imaging
GRASE: gradient and spin echo
G: gradient strength
PNS: peripheral nerve stimulation
RAGE: RApid Gradient Echo
RARE: Rapid Acquisition with Refocusing Echoes
RMSE: root‐mean‐square error
ROI: region of interest
SR: slew rate
SNR: signal‐to‐noise ratio
VASO: vascular space occupancy

## 1 INTRODUCTION

In recent years, advances in high‐field MR systems (≥7 T) and multichannel receive arrays have provided the signal‐to‐noise ratio (SNR) and acceleration necessary to pilot functional MRI (fMRI) at resolutions and timescales previously unreachable. However, a further increase in scan efficiency and spatiotemporal resolution is essential to advance fMRI at the fundamental mesoscopic scale (submillimeter) and timescales well below 1 s. Historically, the development of echo planar imaging (EPI) for fMRI exemplifies how faster and stronger gradient systems have been used to increase scan efficiency as well as resolution.^1^, ^2^, ^3^, ^4^ The performance of current whole‐body gradient systems, however, is limited by both biophysical effects and hardware limits. Consequently, a further increase in scan efficiency and spatiotemporal resolution would require a different approach to gradient system design. Here, compact insertable gradient coils could provide the necessary gradient performance boost while granting ample ease of use and circumventing the physiological limits.^5^

Increased gradient performance primarily assists in reducing the echo‐spacing and thus the readout train length in EPI acquisitions. In particular, high‐resolution fMRI applications such as columnar and laminar level fMRI generally feature long EPI readout trains that constrain scan efficiency and are prone to B_0_ field‐induced geometrical distortions and signal loss.^6^, ^7^ For high‐resolution vascular space occupancy (VASO) fMRI, a fMRI method that has gained a lot interest recently, shorter readouts will also allow shorter echo times and thus less BOLD contamination, thereby increasing VASO fMRI sensitivity.^8^ Furthermore, T_2_* decay during the readout train results in image blurring by degrading the effective voxel size.^9^, ^10^ In addition, arterial spin‐labeling MRI and diffusion‐weighted imaging employing EPI readouts will benefit from the foreseen reductions in echo train length (ETL) and resulting shorter echo times. In summary, shortening the EPI readout train by increased gradient performance will improve high‐resolution fMRI image quality, sensitivity and scan efficiency.

The maximum attainable gradient strength (G) and slew rate (SR) are the two main factors that limit further reductions in the readout train length, and are a function of both the design of the gradient coil and the maximum output of the gradient amplifier (typically approximately in the 1 kV/1 kA range). Generally, higher gradient strength and slew rates are achieved by tailoring the gradient coil design towards a high efficiency (gradient field produced per unit current) and a low inductance (resistance to changing current).^5^, ^11^ Other important factors in the gradient design are Lorentz force management and heating, as the high currents through the conductors that generate the gradient field coincide with strong forces in the magnet of the MRI and resistive losses in the conductors. Consequently, the conductors are embedded in reinforced composites to minimize vibrations and feature a large surface area for cooling, which makes the gradient system bulky and heavy.

The biophysical limit of the gradient system output is predominantly set by the amount of peripheral nerve stimulation (PNS) induced by the switching gradients. In practice, the amount of PNS is determined by the maximum change in the magnetic field, which increases with the slew rate and spatial extent of the gradient fields.^12^, ^13^ Currently, PNS is one of the dominant biophysical factors that limits gradient performance. Local gradient coils or gradient inserts address the problem of PNS by limiting the extent of the gradient field to a smaller region of approximately 30 cm, which can be used to image a specific anatomy, such as the head or extremities. Consequently, these local gradient coils induce less PNS than a whole‐body gradient at the same gradient strength and slew rate.^14^, ^15^ Importantly, decreasing the gradient coil dimensions both increases the coil efficiency and lowers the induction, thereby facilitating higher gradient strengths and slew rates, albeit with a trade‐off in gradient field linearity and thus the extent of the useable imaging field of view (FOV).^16^ By comparison, a clinical MR scanner is often equipped with 40–80 mT/m (strength) and 200 T/m/s (slew) gradient systems. Current state‐of‐the‐art whole‐body gradient systems can produce gradient strengths of up to 300 mT/m with a slew rate of 200 T/m/s.^17^ The current state‐of‐the‐art gradient insert designs allow for gradient strengths of up to 200 mT/m and slew rates of up to 1200 T/m/s.^18^, ^19^

In line with earlier work in 1991 by Turner et al.,^20^ we introduce a compact high‐performance, single‐axis (z‐axis) gradient insert design primarily intended for high‐resolution fMRI. The presented gradient insert is lightweight, takes a short time to install, and operates in synergy with the existing regular whole‐body gradient. The main rationale for a single‐axis approach is that for fMRI generally one gradient axis, that is, along the readout direction, is the most dominant in terms of gradient demand. An exclusive z‐axis gradient design has several benefits: it is relatively easy to design and manufacture; the coil is intrinsically force balanced; and highly effective liquid cooling can be implemented easily using hollow conductors. The main compromise of our single‐axis design is the scan plane in 2D acquisitions, which is limited to the sagittal and coronal plane. This might pose a limitation for direct adoption of this gradient design in clinical practice, where axial scan planes are mainly used. However, for our main application of high‐resolution fMRI, 3D readouts have been widely adopted because of their SNR efficiency and are not limited by our single‐axis approach.^21^, ^22^, ^23^, ^24^ Using recent technology in high‐power gradient amplifiers and by merging the insert with high‐density receiver coil arrays, we demonstrate a practical setup to boost spatiotemporal resolution with EPI.

## 2 METHODS

### 2.1 Design and construction

The design of a gradient insert balances a trade‐off between gradient performance, expected image quality and the physical dimensions of the coil. In particular, for a gradient coil operating along the z‐axis, an increase in gradient efficiency and decrease in inductance requires a reduction of the coil radius and the number of windings, effectively increasing the gradient strength and slew rate.^16^ However, this may reduce the linearity of the gradient field and thus impact the image quality by introducing geometrical distortions.

**FIGURE 1:**
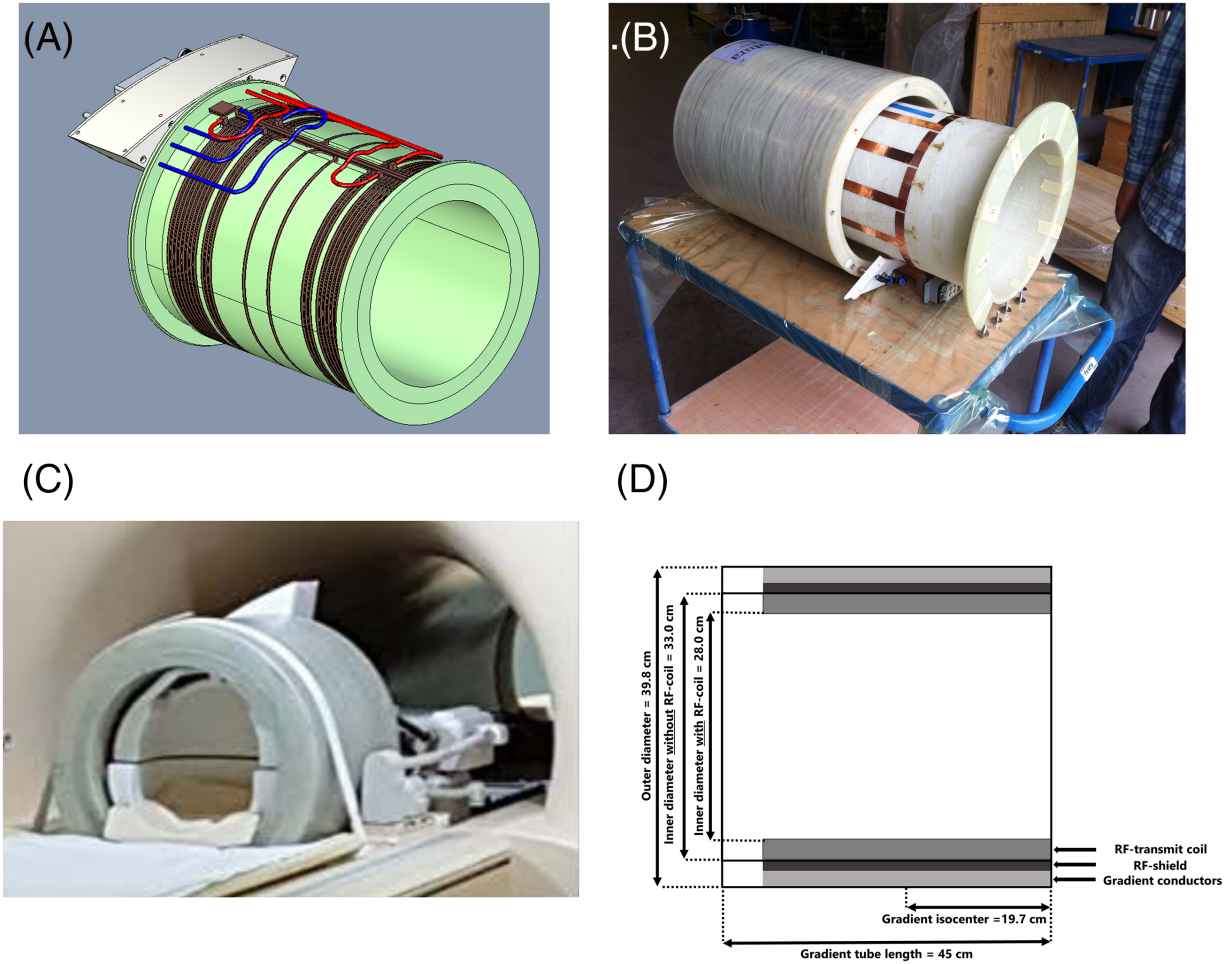
(A) The coil design with cooling conduits in red and blue and conductors in brown. The constructed coil (B) with the integrated birdcage transmit coil; (C) on the patient table showing the inside of the 32‐channe l receive array; and (D) schematic drawing of the gradient insert dimensions

The main objective in our proposed coil design was to be plug and play, that is, the gradient insert should fit in an existing MR system with minimal modifications and be easy to install. Concurrently, active shielding of the gradient insert was omitted, as this added a significant amount of weight and reduced coil efficiency. Furthermore, the small dimensions of the coil would limit the interaction and coupling with materials in the MR bore and cryostat hull, reducing eddy currents. The exact amplitude and time constant of these eddy currents were determined in a simulation study. Hollow conductors were used to allow for active water cooling (Figure 1A). The gradient insert was constrained to an outer diameter of 398 mm and a maximum weight of 45 kg, which means that it fits in the bore of a 7‐T Achieva MRI system (Philips Healthcare, Best, the Netherlands) and may be carried by two people, according to local regulations. The main weight reduction came from the omission of active shielding and the small physical dimensions of the coil. Additionally, the gradient insert included an integrated birdcage transmit coil and is able to house a commercially available 32‐channel receive head coil (Nova Medical, Wilmington, MA, USA). The integrated transmit coil is mounted on an interchangeable tube (Figure 1B), which provides easy access for maintenance and facilitates the use of differently tuned RF transmit coils in the case of different field strengths or nuclei. Both the gradient and transmit coil tube were made from a fiberglass epoxy resin.

In our design, we aimed for a coil with an efficiency of 0.30 mT/m/A and a maximum inductance of 110 μH, while allowing a maximum deviation from gradient linearity of 5% over a spherical region of 16‐cm diameter. Importantly, this range of the linear region will allow brain imaging with only a limited amount of geometrical distortion. The optimal winding positions were determined by means of a genetic algorithm from the Global Optimization Toolbox in MATLAB (MathWorks, Natick, MA, USA). This optimization was performed for a winding configuration of 12 Maxwell pairs (24 windings in total), which was the minimum number of windings needed for our desired efficiency. Additionally, the diameter of the windings was limited to 352 mm to fit the RF coils.

### 2.2 Gradient insert specifications

The gradient insert was constructed by Futura Composites B.V. (Heerhugowaard, the Netherlands) according to the design described above, which is also shown in Figure 1 (the exact winding positions can be found in Figure S1 and Table S1 in the supporting information). The final inner diameter of the gradient insert when embedded in epoxy was 28 cm including the RF transmit coil (33 cm without the RF transmit) and the distance from the shoulder opening to the isocenter was 19.7 cm. The expected electrical properties were calculated based on material properties and the dimensions of the conductors, and validated with an LCR meter (Keysight Technologies, Santa Rosa, CA, USA) at DC and at 1 kHz, as shown in Table 1. In simulation, the dominant eddy current was found to feature a first‐order spatial variation in the z‐direction, an amplitude of 1% of the applied gradient and a time constant of 0.5 s. For the measurements in this work, this eddy current was not corrected. A dedicated amplifier (Prodrive Technologies, NG500) was used to drive the gradient insert at a maximum of 940 V and 630 A. Theoretically, this would yield maximum values of gradient strenght and slew rate of 200 mT/m and 2642 T/m/s, respectively. Practically, the maximum step response of the amplifier of 4 A/μs limits the maximum slew rate to 1300 T/m/s. The use of a dedicated amplifier also reduced the installation time as the amplifier is permanently tuned for the load of the gradient insert and does not need to be retuned to also accommodate the load of the whole‐body gradients.

**TABLE 1:** Expected and measured electrical properties of the designed gradient insert

	Expected value	Measured value (DC)	Measured value (@1 kHz with cable and filter panel)
Resistance	22.3 mΩ	18.5 mΩ	81 mΩ
Inductance	108.7 μH	109.2 μH	113 μH

### 2.3 Plug‐and‐play gradient installation

The dedicated gradient amplifier and cooling cabinet were permanently installed in the technical room to facilitate the plug‐and‐play installation of the gradient insert. Additionally, the installation of these devices required a high‐power mains connection, an additional gradient filter and space for cooling lines in a waveguide. The gradient cables and cooling lines were connected to the devices and permanently stored in the scanner room to allow for easy access. During the plug‐and‐play installation, the gradient insert could be installed by shifting the receiver array out of the standard RF transmit coil, and replacing the RF transmit coil by the gradient insert, which was equipped with its own transmit coil. This required the repositioning of the receiver array and reconnection of two RF transmit lines, one detuning line, and connecting one plug to the gradient interface to provide the gradient waveform to the dedicated amplifier. In addition, the software patch that enabled driving the gradient insert required one reboot of the scanner spectrometer, and the gradient amplifier had to be switched on. This installation procedure took less than 15 min and a typical timetable can be found in Table 2. The setup can also be fitted with an fMRI mirror system for presenting visual stimuli (Figure S2/S3). In this work, no cooling was used as the gradient insert was driven at low duty cycle for all experiments.

**TABLE 2:** Typical timetable of the installation procedure of the gradient insert

	Time, min
Restarting and patching software	5
Removing standard RF transmit coil	2
Installing gradient insert on system	5
Turning on gradient amplifier	2
Total time	14

### 2.4 Peripheral nerve stimulation

PNS measurements were performed to assess the gradient strength and slew rate permitted by human physiology. Here, a range of slew rates and gradient strengths was probed by operating the gradient insert coil using different trapezoidal pulse shapes. Five different rise times were used, of 25, 50, 153, 400 and 600 μs, and the gradient amplitude was varied up to the hardware limits, using step sizes of 10–15 mT/m. At each rise time and gradient amplitude, four pulse trains of 50 ms were applied, with a pause of 1 s between consecutive pulses^25^, ^26^, ^27^ and a plateau length of 350 μs. The rise times were executed in random order, unknown to the subject. For each rise time the gradient amplitudes were executed in ascending order. After each pulse train, the subjects indicated if they had experienced PNS, and indicated the intensity of PNS sensations on a scale of 1–5 (1 = very mild, 5 = painful). Here, a PNS sensation of 2 was defined as a clear sensation of PNS. After the measurements the volunteers were asked for the nature of the PNS sensations. The data points with very mild PNS sensation (1 on the scale of 5) were used to fit a linear PNS threshold curve,^28^ yielding the PNS parameters ΔG_min_, ΔSR_min_ and τ_c_. Here, ΔG_min_ is the minimal peak‐to‐peak change in gradient amplitude that induces PNS at an infinitely short rise time, ΔSR_min_ is the minimum slew rate needed to induce PNS at an infinite rise time, and τ_c_ is the chronaxie, which is a tissue‐specific parameter defined as the ratio of ΔG_min_ and ΔSR_min._ A logistic regression was used to estimate the gradient amplitude needed for stimulation for rise times where not all subjects experienced PNS.^15^ The measurements were performed in six volunteers (two females and four males, age 25–45 years). Informed consent was given by all volunteers in accordance with the Institutional Review Board of the UMC Utrecht.

### 2.5 Simulations

The minimum echo‐spacing achievable for a given resolution and FOV is determined by the maximum gradient strength and slew rate that can be used in practice. The maximum readout bandwidth provided by the scanner (1 MHz) poses a potential limiting factor for the maximum gradient strength useable, because the combination of gradient strength and bandwidth directly determine the maximum FOV possible through the following equation:

(1) $$\mathrm{FOV}=\left(\text{2π}\cdot \mathrm{BW}\right)/\left(\gamma \cdot G\right),$$

where *BW* is the readout bandwidth of the scanner, γ the gyromagnetic ratio of hydrogen and *G* the gradient strength in T/m. Using Equation 1, the effect of maximum bandwidth and gradient strength on FOV size was simulated for a range of bandwidths of 0.1–3 MHz and gradient strengths of up to 400 mT/m. This allowed us to investigate the limitations imposed by the current scanner bandwidth of 1 MHz, and the potentially useable gradient strengths with future scanner and gradient hardware.

The effect of the increased gradient performance (strength and slew rate) on echo‐spacing was simulated for a single‐shot EPI readout with a maximum slope sampling of 80%, a FOV of 50–256 mm and a resolution of 0.2–2 mm. Importantly, the maximum slope sampling possible was determined by the sampling dead time introduced by the phase‐encoding gradient. Additionally, the maximum bandwidth and minimum rise time were set to 1 MHz and 50 μs, respectively, in correspondence with current scanner limits at our site. The shortest echo‐spacing was determined by considering all possible combinations of gradient strength and slew rate for each combination of FOV and spatial resolution in the readout direction. The resulting echo‐spacing was compared with the shortest echo‐spacing possible using the maximum gradient strength and slew rate of the existing whole‐body gradient coil, which were 40 mT/m and 200 T/m/s, respectively.

Most gradient inserts feature all three gradient axes, which potentially yields a larger reduction in echo‐spacing compared with the proposed single‐axis gradient insert and wider applicability. To investigate the potential benefit in performance, the aforementioned simulations were repeated with a theoretical setup featuring all three axes with the same performance as the proposed single‐axis setup (G = 200 mT/m, SR = 1300 T/m/s). Importantly, in these simulations a minimum rise time of 1 μs was used instead of 50 μs to allow for a wider range of useable slew rates. Here, the minimum rise time was defined as the shortest duration of a ramp at the maximum slew rate of 1300 T/m/s. The additional performance gain of a three‐axes design was quantified by comparing the minimum achievable echo‐spacing with the proposed single‐axis design.

### 2.6 Gradient field and performance characterization

Characterization of the gradient field generated by the designed gradient insert was performed in three steps. First, the field distribution of the gradient insert was measured, which allowed us to determine the efficiency and region linearity. Next, the measured field distribution was used to correct the image distortion caused by nonlinearities in the produced gradient field. Finally, EPI was performed to study the effect of increased gradient performance on ETL and image quality.

### 2.7 Field distribution measurements

The field distribution was measured using a series of B_0_‐field maps acquired with the whole‐body gradients of the system. The B_0_‐field maps were derived from 3D gradient echo sequences with ΔTE = 0.5 ms, in‐plane resolution = 3.75 x 3.75 mm^2^, FOV = 480 x 480 mm^2^, slice thickness = 3.75 mm, number of slices = 64, flip angle = 2°, TR = 4.0 ms and TE_1_ = 1.6 ms. Concurrently, a constant current was provided to the gradient insert, hence superimposing the field distribution of the gradient insert on the B_0_‐field maps. Different parts of the field distribution were sampled by varying the position of a phantom inside the gradient insert. Here, the phantom consisted of a 30‐cm (8‐cm radius) long bottle of saline water. Additionally, the acquisition at each phantom position was repeated for different current amplitudes (0.5 and 1 A) and polarity of the driving current. All field measurements were then combined to form the final field distribution, which was compared with the theoretical field distribution produced by the known winding configuration.^29^

### 2.8 Geometrical distortion

The partially nonlinear field of the gradient insert led to geometrical distortions during imaging, which were assessed using a geometry phantom. The phantom featured grid points that were arranged in a 15 x 15 cm^2^ grid with 2.5‐cm spacing between the grid points. For the gradient insert, an image‐based geometrical correction^30^ was used to correct the geometrical distortions where possible. In this method, the spatial displacement caused by the nonlinear field is corrected in image space by applying a coordinate transform, which uses the difference between the used encoding fields and the ideal encoding field to map the data to their true position. The whole‐body acquisitions were corrected using the scanners’ geometry correction, which utilizes spherical harmonics up to the fourth order.

A 3D gradient echo sequence was used with in‐plane resolution = 1.5 x 1.5 mm^2^, FOV = 500 x 500 mm^2^, slice thickness = 1 mm, number of slices = 15, flip angle = 45°, TR = 16.21 ms and TE = 6.99 ms. Furthermore, a reference for the geometrical distortion was obtained by repeating the same acquisition with the whole‐body gradients. The geometrical distortion was quantified by comparing the positions of grid points in the reference acquisition and the gradient insert acquisitions. Here, the position of a grid point was defined by its centroid. Grid points from different acquisitions were quantified as overlapping in space when the distance between grid points was less than one voxel. The average offset and root‐mean‐square error (RMSE) were used as error measures. An in vivo example of the geometrical distortion was also acquired and can found in Figure S4.

### 2.9 Echo planar imaging

The effect of increased gradient performance on echo‐spacing and geometrical distortion was studied using a 2D single‐shot gradient echo EPI with an in‐plane resolution = 1 x 1 mm^2^, FOV = 192 x 192 mm^2^, slice thickness = 3 mm, flip angle = 90°, slope sampling = 80% and TR = 1000 ms. This sequence was repeated for three different combinations of slew rate in T/m/s and gradient strength in mT/m: SR/G = 200/40, 800/96 and 1290/112. Additionally, the whole‐body gradients were driven at maximum performance (G = 40 mT/m, SR = 200 T/m/s) as a benchmark. A 10‐cm diameter sphere containing saline water was used as a phantom. The geometrical distortion was quantified by determining the overlap between the measured and theoretical sphere area. The reduction in distortion achieved by the gradient insert with respect to the whole‐body gradient set was calculated as follows:

(2) $$\left({\text{Residual distortion}}_{\text{gradient insert}}-{\text{Residual distortion}}_{\text{whole}-\text{body}}\right)/{\text{Residual distortion}}_{\text{whole}-\text{body}}$$

Additionally, no shimming was applied, as this makes the reduction in echo‐spacing the only source of improvements in the geometrical distortion.

To showcase the benefit of the increased gradient performance and that the setup supports presentation of visual stimuli, a simple visual BOLD fMRI experiment was performed. BOLD fMRI was performed on the visual cortex using a 2D single‐shot gradient echo EPI with an in‐plane resolution = 1 x 1 mm^2^, FOV = 192 x 192 mm^2^, slice thickness = 3 mm, a coronal scan plane, flip angle = 90°, TE/TR = 25/1000 ms and an acceleration factor of R = 2, first‐order B_0_ shimming and 100 volumes were acquired. For this experiment, the gradient insert was driven at SR = 616 T/m/s and G = 85 mT/m. Note that this is not the maximum attainable gradient slew rate and strength possible with the gradient insert setup, as this was chosen to limit the duty cycle and allows for scanning without cooling. The same acquisition was repeated using the whole‐body gradients. This scan featured the same imaging parameters as the scan with the gradient insert, except for a longer echo time of 40 ms, which was limited by the gradient performance of the whole‐body gradients. The setup and subject were fitted with an fMRI mirror system and prism glasses, respectively (Figure S2/S3). The subject was given a visual task using a 20‐s on/off block design (manually triggered), where a uniform gray screen was alternated with a flickering checkboard at 8 Hz. BOLD activation maps (z‐statistics) were obtained using FEAT (FSL, FMRIB OXFORD).^31^ Informed consent was given by the subject in accordance with the Institutional Review Board of the UMC Utrecht.

Acoustic noise measurements were performed for all the EPI scans in this section using a microphone (Behringer ecm8000) placed at a position mimicking the position ear of a subject. The audio waveforms were recorded and processed in MATLAB and the setup was calibrated using a sound calibrator (type 4231, Brüel & Kjær, Denmark).

## 3 RESULTS

### 3.1 Peripheral nerve stimulation

**FIGURE 2:**
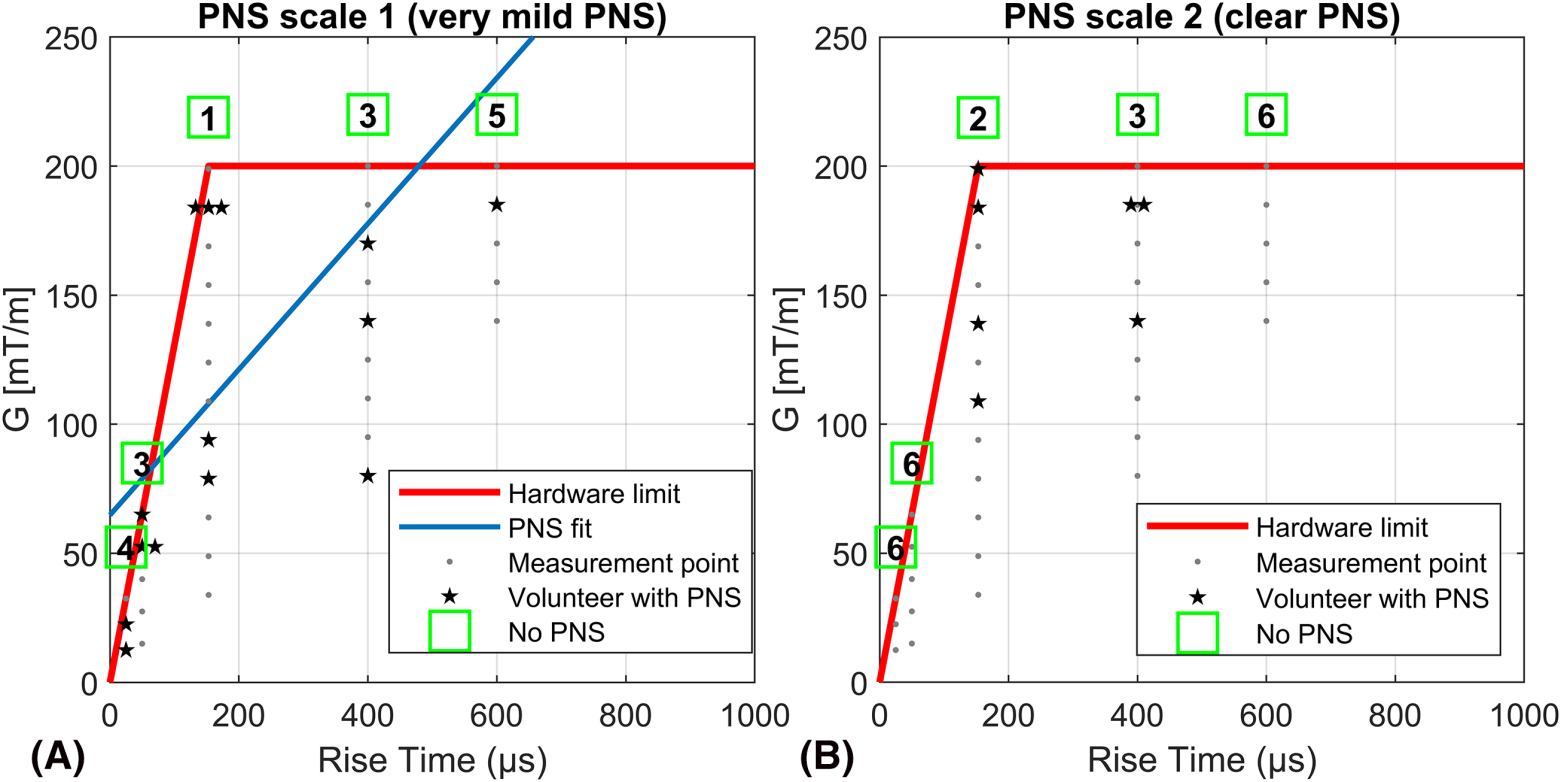
Results of the peripheral nerve stimulation (PNS) measurements for the six volunteers experiencing very mild (A) and clear sensation of PNS (B). Here, each measured combination of gradient strength and rise time is indicate by the dots. The star‐shaped markers indicate combinations of gradient strengths and rise time that resulted in noticeable PNS for one volunteer. The number of star‐shaped markers indicate the number volunteers experiencing noticeable PNS. The numbers in the green boxes indicate the number of volunteers that did not experience any noticeable PNS at a particular rise time

The results of the PNS measurements are summarized in Figure 2, which shows the data points for subjects scoring a PNS sensation as very mild (1/5) and clear PNS (2/5). One subject reported moderate PNS (3/5) at a rise time of 400 μs and an amplitude of 185 mT/m. The fit of the PNS threshold yielded values for the PNS parameters of ΔSR_min_ = 282.2 ± 80.7 T/m/s, ΔG_min_ = 64.8 ± 25.6 mT/m and τ_c_ = 229.7 ± 112.0 μs. The large uncertainties in the PNS parameter are due to the relatively small sample size of the PNS measurements. In the cases that PNS was experienced, the sensation usually occurred in the face or cheeks with one subject reporting a sensation in the neck and shoulder area.

### 3.2 Simulations

**FIGURE 3:**
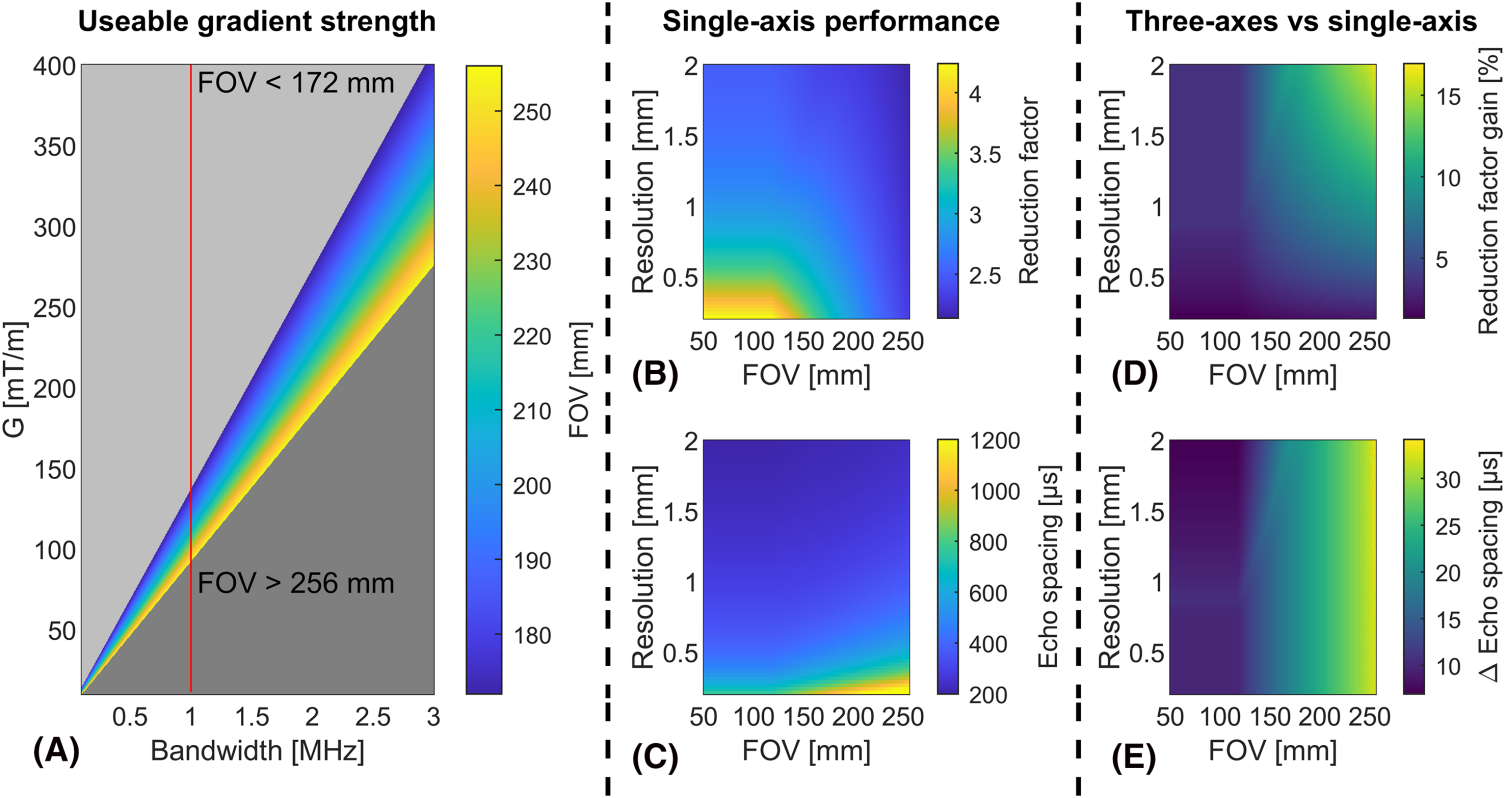
(A) Simulation results for the maximum field of view (FOV) possible for different combinations of bandwidth and gradient strength. The red line indicates the maximum bandwidth used for the performed experiments in this study; (B) simulation results for the maximum reduction of the echo‐spacing for different combinations of FOV and resolution. The acceleration factor was determined with respect to the maximum performance of our whole‐body gradient set (G = 40 mT/m, SR = 200 T/m/s); (C) the minimum echo spacing achievable using the gradient insert for different combinations of FOV and resolution; (D) the additional reduction factor achievable using three high performance gradient axes (G = 200 mT/m, SR = 1300 T/m/s); and (E) the additional decrease in echo‐spacing achievable when using three high performance gradient axes (G = 200 mT/m, SR = 1300 T/m/s)

The simulation results in Figure 3A show that the maximum acquisition bandwidth of 1 MHz of the MRI system limits the maximum FOV achievable in the readout direction when increasing the gradient strength. The maximum useable gradient strength is limited to 110–120 mT/m for FOVs encompassing the whole brain (192–256 mm). For a gradient strength of 200 mT/m, the maximum FOV was 117 mm. Doubling the maximum gradient strength to 400 mT/m yielded a maximum FOV of 58.7 and 176 mm for bandwidths of 1 and 3 MHz, respectively.

Figure 3B shows the reduction in echo‐spacing for different combinations of spatial resolution and FOV when comparing the gradient insert with the body gradient setup. For FOVs smaller than 252 mm, all the simulated combinations of FOV and resolution showed at least a twofold reduction in echo‐spacing when using the gradient insert. The minimum reduction in echo‐spacing was a factor of 2.1 for a FOV of 256 mm and a resolution of 2 mm. The maximum reduction in echo‐spacing by a factor of 4.2 was observed for a resolution of 0.2 mm and a FOV of 50–127 mm. Furthermore, we found a maximum reduction in echo‐spacing of a factor of 2.6 for the acquired EPI acquisition obtained at a resolution of 1 x 1 mm^2^ and a FOV of 192 x 192 mm^2^. In the simulations, the maximum reduction was achieved for a gradient strength of 122 mT/m and a slew rate of 1300 T/m/s.

Figure 3C shows the echo‐spacing achievable with the gradient insert for different combinations of spatial resolution and FOV. Here, the echo‐spacing was found to increase for higher resolutions. We found an expected echo‐spacing of 325 μs for the aforementioned EPI acquisition with G =112 mT/m and SR = 1290 T/m/s.

The comparison between the theoretical three‐axes setup and our single‐axis gradient insert is shown in Figure 3D,E. In Figure 3D, the three‐axes setup shows a maximum additional reduction of echo‐spacing of 17% for a FOV of 255 mm and a resolution of 2 mm. For resolutions of 1 mm and higher, the maximum additional reduction of echo‐spacing was found to always be less than 10%, irrespective of the FOV.

Figure 3E shows the difference in achievable echo‐spacing for the three‐axes and single‐axis setups. The effect of the three‐axes setup on reducing echo‐spacing was found to be proportional to the FOV. The additional decrease in echo‐spacing ranged from 30 μs for FOVs larger than 240 mm to less than 10 μs for FOVs smaller than 118 mm.

### 3.3 Field distribution measurements

**FIGURE 4:**
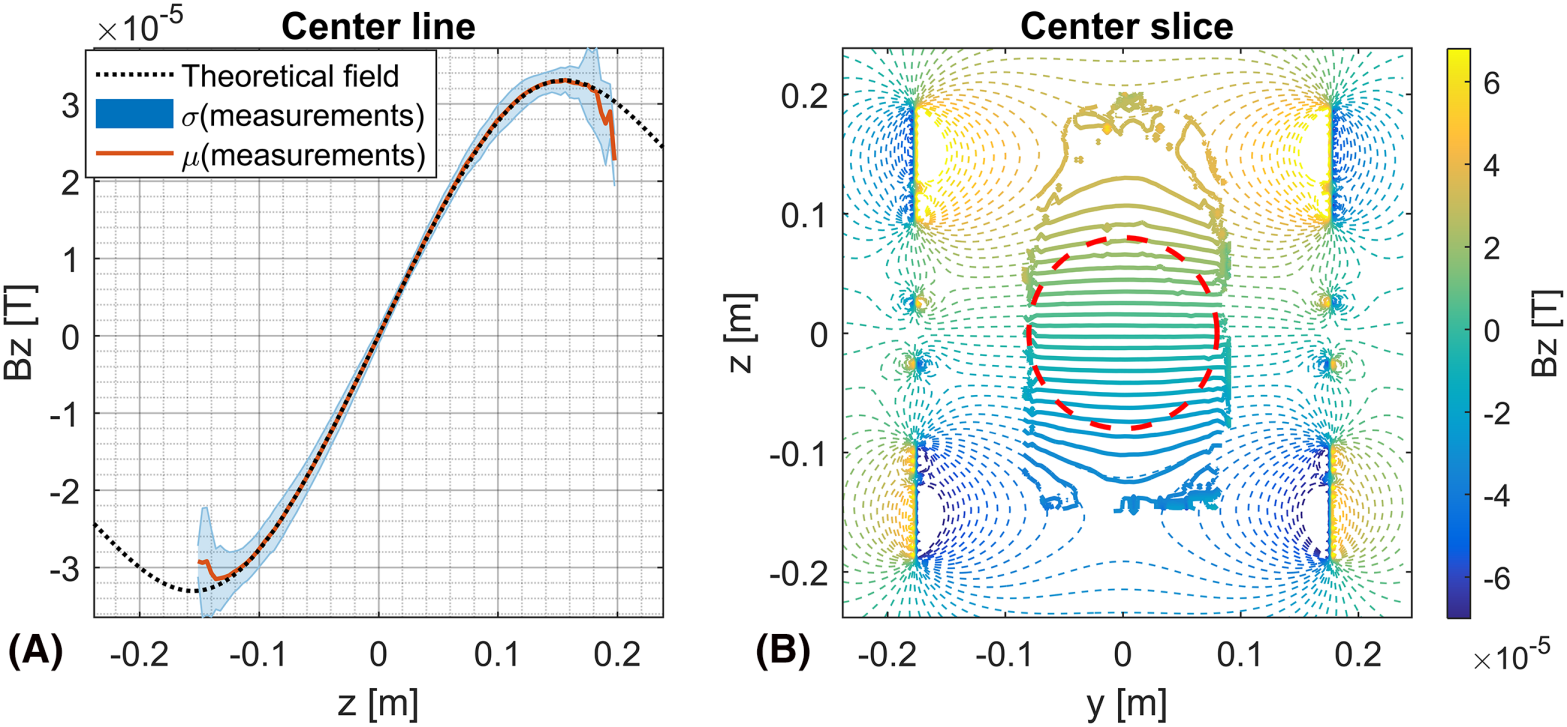
Theoretical and measured field distributions for a driving current of 1 Ampère. (A) Across a line through the gradient insert. (B) In a sagittal slice through the gradient insert. Here, the red sphere indicates the 16‐cm spherical region with ≤5% deviation from linearity. Here, the dashed lines indicate the theoretical field as obtained from the design, and the solid lines show the measured field distribution obtained from the B0‐maps

The measured and predicted field distributions are shown in Figure 4. Figure 4 showcases a close correspondence between the theoretically expected and measured field distribution. Here, a 16‐cm region of linearity was found for a maximum field deviation of 5% and a 22.7‐cm region of linearity was found for a maximum field deviation of 15%. Notably, the difference between the measured and expected field distribution was lowest in the region of linearity, while the largest deviations from the expected field distribution were observed at the fringes of the measured volume. Additionally, the measured efficiency of 0.32 mT/m/A corresponded to the theoretically predicted efficiency (0.32 mT/m/A).

### 3.4 Geometrical distortion

**FIGURE 5:**
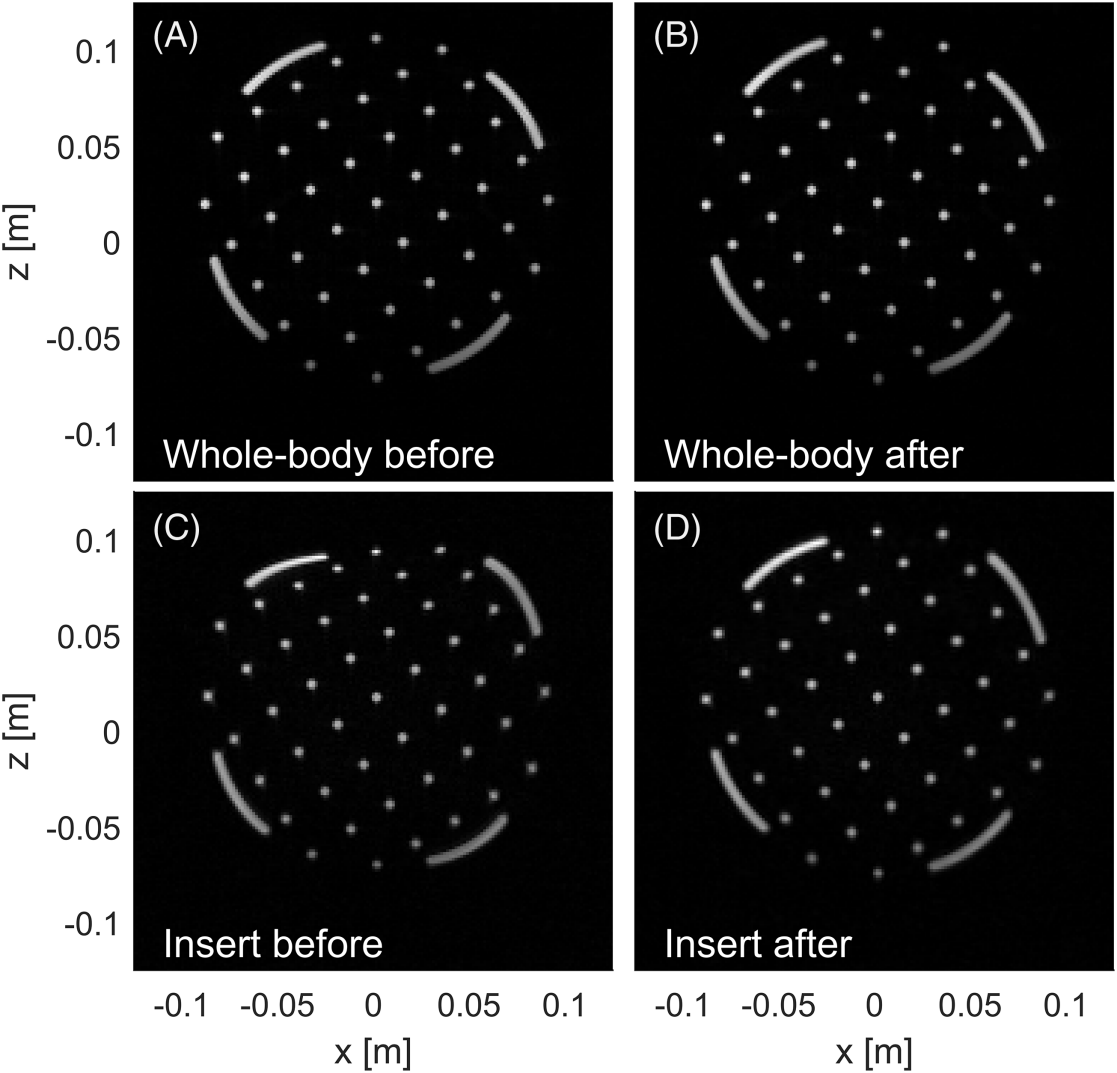
Comparison of the geometric distortion produced by (A) the whole‐body z‐gradient before the geometry correction, (B) the whole‐body z‐gradient after the geometry correction, (C) the gradient insert before geometry correction and (D) the gradient insert after geometry correction. Here, the whole‐body system was driven at G = 5 mT/m and  SR = 8 T/m/s and gradient insert at G = 23.5 mT/m and SR = 74 T/m/s

The acquisitions performed on the geometry phantom with both the whole‐body z‐gradient and the gradient insert are shown in Figure 5. Figure 5A shows the uncorrected whole‐body gradient acquisition, which featured small geometrical distortions at the edge of the phantom. Figure 5B shows the corrected whole‐body z‐gradient acquisition, which was used as a reference to which the other corrections were compared. The maximum distance between a grid point and the gradient isocenter was 10 cm, thus allowing us to probe the distortion for a target FOV of 20 cm.

Without geometry correction, the image acquired with the gradient insert (Figure 5C) shows large geometrical distortions, with up to a 1‐cm mismatch to the reference, an average offset of 1.7 mm and an RMSE of 2.2 mm. After applying the geometry correction (Figure 5D), the average offset of the grid points was 0.78 mm (RMSE = 1.2 mm). When only considering points inside the linear region (target FOV = 16 cm), 37/39 points (94.8%) overlapped with the reference with an average offset of 0.55 mm (RMSE = 0.78 mm).

### 3.5 Echo planar imaging

**FIGURE 6:**
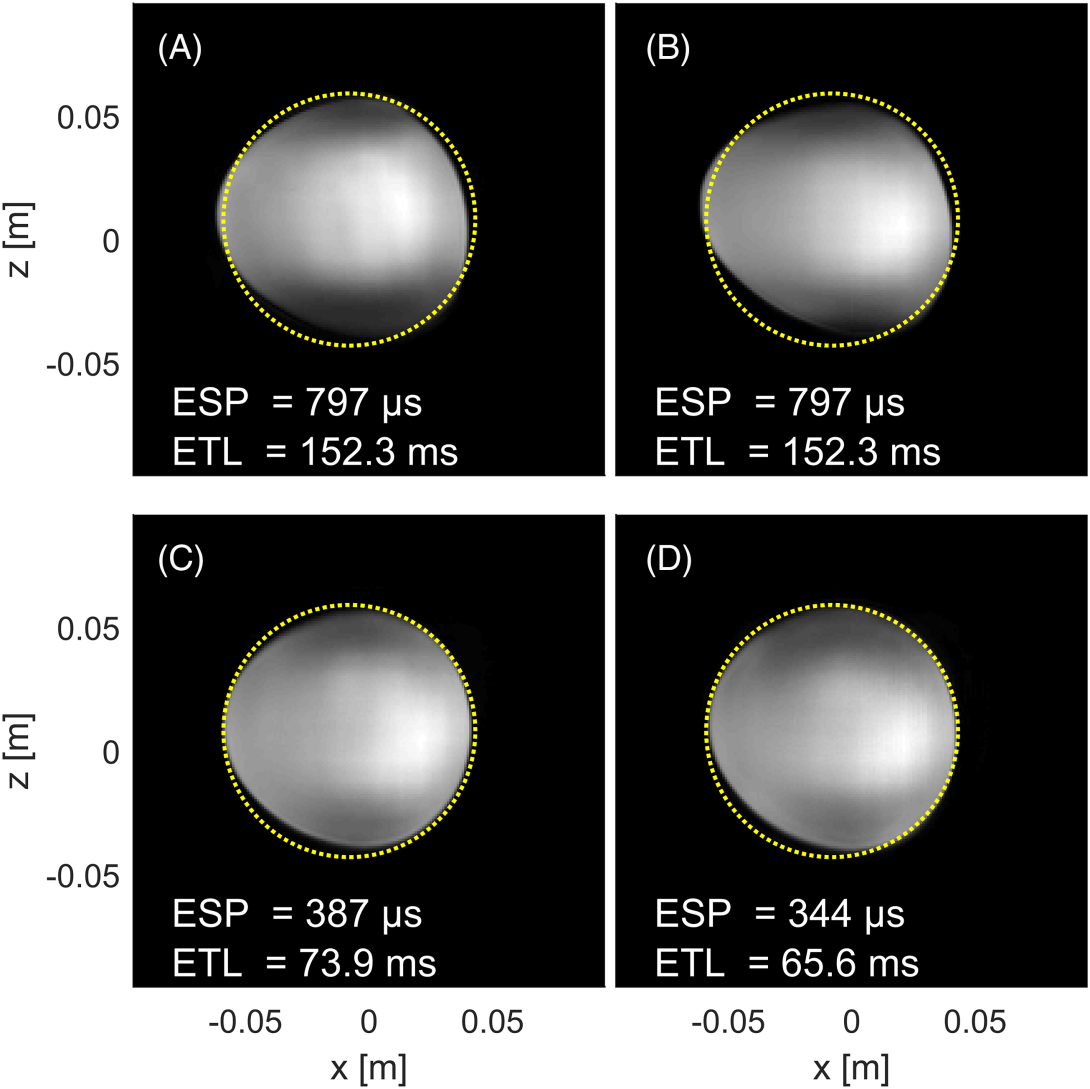
The effect of different combinations of slew rate and gradient strength on the echo‐spacing (ESP) and geometrical distortions apparent in an echo planar imaging (EPI) acquisition (resolution = 1 x 1 mm^2^; field of view = 192 x 192 mm^2^) for (A) the whole‐body z‐gradient used at G = 40 mT/m and SR = 200 T/m/s, (B) the gradient insert used at G = 40 mT/m and SR = 200 T/m/s, (C) the gradient insert used at G = 96 mT/m and SR = 800 T/m/s, (D) the gradient insert used at G = 112 mT/m and SR = 1290 T/m/s. Note the decrease in echo‐spacing and geometrical distortion with increased gradient performance. The yellow circle indicates the outline of the physical sphere. ETL, echo train length

Figure 6 shows the influence of gradient performance on the geometrical distortion and echo‐spacing in EPI acquisitions at 7 T. For the same gradient performance, the acquisitions with whole‐body gradients and the gradient insert showed similar imaging results because both featured a distorted sphere (Figure 6A,B), which overlapped for 94% (Figure 6A) and 91% (Figure 6B) with the known area of the spherical phantom. The increased gradient performance of the insert resulted in a decrease in geometrical distortion (Figure 6C,D), as illustrated by the 96% (SR/G = 800/96) and 98% (SR/G = 1300/112) overlap with the ground truth geometry of the phantom, which equated to 4% and 2% residual distortion, respectively. With respect to the whole‐body gradient image, we observed a 67% reduction in distortion.

A minimum echo‐spacing of 344 μs was obtained for a gradient strength of 112 mT/m and a slew rate of 1290 T/m/s. This translated to a factor of 2.3 reduction in echo‐spacing compared with the maximum performance of the whole‐body gradient system. Notably, we observed a similar decrease in echo‐spacing to 387 μs for a lower gradient performance with G = 96 mT/m and SR = 800 T/m/s, respectively, which visually yielded similar geometrical distortion to that obtained with higher gradient performance. The measured reduction in echo‐spacing approached the theoretical maximum performance gain for the used FOV and resolution found in the simulations (Figure 3B,C), although the measurement featured a slightly lower gradient strength (112 vs. 122 mT/m).

**FIGURE 7:**
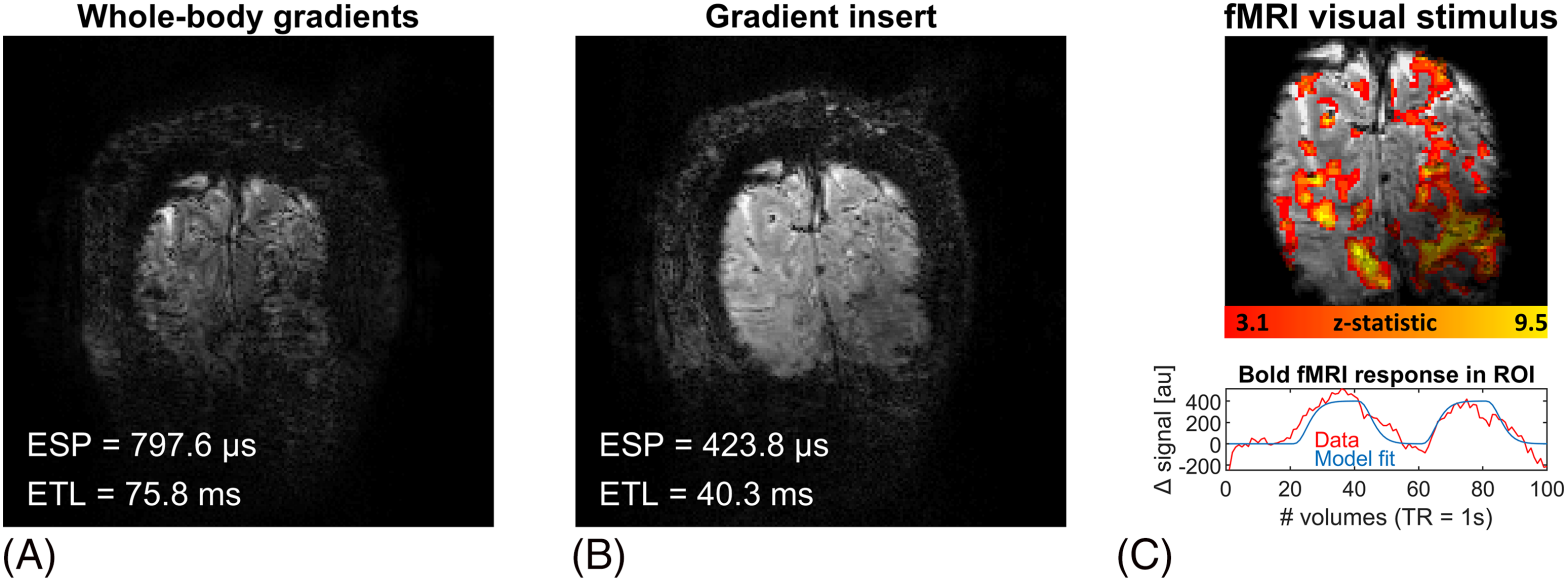
Results of the in vivo example for a single‐shot echo planar imaging (EPI) acquisition of the primary visual cortex (spatial resolution = 1 x 1 mm^2^; field of view = 192 x 192 mm^2^) for (A) the whole‐body z‐gradient used at G = 40 mT/m and SR = 200 T/m/s. Note the substantial geometric distortion and signal loss. (B) The gradient insert used at G = 85 mT/m and SR = 616 T/m/s. Note the reduction in distortion observed from the shorter echo train achieved by the gradient insert. (C) The same EPI readout used for a simple visual BOLD functional magnetic resonance imaging (fMRI) experiment: (top) the resulting BOLD activation pattern in the visual cortex; (bottom): the average change in signal in the activated regions during the visual task. ESP, echo‐spacing; ETL, echo train length; ROI, region of interest

Figure 7 shows the in vivo example of the BOLD 2D EPI fMRI experiment using a simple visual task. Substantial reductions in echo‐spacing of 727.6 to 424 μs and in ETL of 75.8 to 40.3 ms were obtained when using the gradient insert. Also, the minimal attainable echo time decreased from 40 to 21 ms. These reductions resulted in notably less geometrical distortions and less signal loss (Figure 7A,B). Figure 7C shows the BOLD fMRI activation pattern evoked by the visual task using the gradient insert setup. The activation pattern was observed throughout the imaged primary visual cortex, showcasing that visual stimuli can be adequately presented. Note that the gradient insert setup was not driven at maximum performance for this in vivo example.

The acoustic noise production of the gradient insert during the EPI acquisitions was found to be at a similar level to the whole‐body gradient system. For the highest gradient performance (G = 112 mT/m and SR = 1290 T/m/s), the gradient insert produced an A‐weighted equivalent (LA_eq_) of 112 dB and a peak sound pressure level (LC_peak_) of 124 dB, which was comparable with the LA_eq_ = 112 dB and LC_peak_ = 123 dB of the whole‐body gradients driven at maximum performance (G = 40 mT/m and SR = 200 T/m/s). Sound levels of the other EPI acquisitions can be found in Table S2.

## 4 DISCUSSION

This work describes the design of a novel lightweight gradient insert that can be installed using a plug‐and‐play approach within 15 min. A fivefold increase in gradient strength and a sixfold increase in slew rate were achieved compared with a standard whole‐body gradient system, while producing small but correctable geometric distortions in a spherical region of 16‐cm diameter. Furthermore, simulations and measurements showed at least a factor of 2 reduction in echo‐spacing (and thus readout train length) for the FOVs and resolutions relevant for state‐of‐the‐art functional brain imaging.

The constructed coil met or exceeded the minimum design specifications for the efficiency (measured 0.32 vs. designed 0.3 mT/m/A), inductance (measured 109.2 vs. designed 110 μH) and region of linearity (<5% deviation in a 16‐cm diameter spherical region). The coil design omitted the precondition of a large linear region to enforce a low inductance coil and thus fast switching capabilities. Accurate knowledge of the field distribution was used in the geometrical distortion correction to mitigate this trade‐off between performance and image fidelity. Here, the resulting distortions in the nonlinear region (>16 cm) were successfully corrected to within 6% of the reference image fidelity. The majority of residual distortions was located outside the linear region and were likely caused by concomitant fields and residual B_0_ inhomogeneities after B_0_ shimming.

The small size and low weight of the coil resulted in limited modifications to existing scanner hardware and short installation times. The installation procedure can be described as being similar to setting up a dedicated RF head coil on the patient table. Thus, the coil can be positioned between scan sessions, such that the availability of the MR system and the daily workflow of head and body imaging are largely unaffected. With this plug‐and‐play feature, the coil provides an interesting alternative to existing gradient inserts and dedicated head MR scanners.^32^ Existing gradient inserts can feature considerable modifications to the scanner hardware (e.g. removal of patient tubes and RF coils), resulting in installation times of up to 1 day.^18^, ^33^

The single‐axis design was aimed at a reduction in echo‐spacing and thus ETL in EPI acquisitions. In the presented simulations, the echo‐spacing reduction with respect to the whole‐body gradient was found to be at least a factor of 2 for all combinations of resolution and FOVs. Notably, the largest reductions in echo‐spacing (up to a factor of 3) were found for submillimeter resolutions, paramount for mesoscopic scale MRI.^7^, ^34^ In our measurements, the coil achieved an echo‐spacing reduction by a factor of 2.3, corresponding to an echo‐spacing of 344 μs for the used EPI readout. This is in close accordance with the simulations. Furthermore, the corresponding reduction in ETL reduced the geometrical distortions by 67% compared with the whole‐body gradient set. The decreased echo‐spacing results in a decrease in SNR due to the higher receiver bandwidth in combination with a higher gradient strength. We found this SNR penalty to be offset by the benefits from shorter echo‐spacing scans and the extra available SNR when performing 3D EPI readouts.^10^ A further reduction in echo‐spacing can be achieved by removing the phase‐encoding blips, which effectively results in the original proposed EPI sequence by Mansfield.^1^ The omission of the phase‐encoding blips will result in more time‐efficient sampling by allowing full ramp sampling during the readout EPI gradient lobes. An additional benefit here is also a substantial decrease in acoustic noise.^35^

Our single‐axis approach limits the slice orientations, for which a performance boost is obtained compared with three‐axes gradient inserts. Specifically, the maximum echo‐spacing reduction is achieved only when using either coronal or sagittal slices, as the readout train is then oriented in the z‐direction. In terms of echo‐spacing reduction, our simulations show that adding additional high‐performance gradient axes to our gradient insert does not lead to a significant additional reduction in echo‐spacing for a conventional EPI readout. For high‐resolution EPI (≤1 mm), the echo‐spacing was further reduced by 10%, which is small compared with the greater than 200% reduction already achieved by the single‐axis design in synergy with a whole‐body system. Additionally, the small performance boost from adding additional gradient axes should be compared with the engineering challenges involved, such as cooling of the extra axes, arcing due to high voltage differences between axes and the substantial increase in size and weight.^36^ It should be noted, however, that other applications using non‐Cartesian readouts (e.g. spiral readouts) and diffusion MRI do benefit from the availability of three high‐performance gradient axes.^18^, ^27^, ^33^

Factors that limit the current maximum achievable gradient performance are predominantly biophysical effects but also the available hardware. In terms of bioeffects, PNS and acoustic noise are generally considered the main limiting factor for increasing gradient performance. Previous studies have found that for coil designs with a similar gradient performance but with larger encoding fields compared with our insert, and therefore more likely to induce PNS, PNS was low and practically not restricting.^18^, ^26^ Our PNS measurements suggest similar behavior for our gradient insert with the majority of occurrences of a clear sensation of PNS around the hardware limits of the gradient insert. However, not all subjects experienced PNS and a larger sample size or a more powerful amplifier is needed to yield a more accurate PNS threshold. The gradient insert and whole‐body gradients showed similar acoustic noise levels for the EPI sequences used in this paper. Consequently, the same hearing protection can be used for these sequences. In terms of hardware, we identified the available bandwidth as the main limiting factor, because it constrains the FOV. Higher bandwidth spectrometers or perhaps parallel imaging in the readout direction are needed to use the maximum available gradient strength for a wider range of attainable FOVs. Importantly, this hardware limit might prove to be an important design consideration for future gradient insert designs aimed at even higher gradient strengths (>200 mT/m), as our simulations show that whole‐brain imaging at these high gradient strenghts is unfeasible unless this bandwidth issue is addressed.

Future work on improving image quality will explore dedicated reconstruction approaches and different modes of driving the gradient coil. In terms of reconstruction, image quality can benefit from nonuniform fast Fourier transform or iterative approaches to correct for the inherent nonlinear signal encoding from the gradient insert.^37^, ^38^, ^39^ Other encoding imperfections, due to eddy currents, B_0_ offsets and concomitant fields, can also be incorporated in the reconstruction by field camera characterization, which previously has been shown to greatly improve image quality.^40^ In terms of alternative driving modes, the gradient insert can in principle work in synergy with the whole‐body gradient as an independent fourth axis. The additional z‐axis can potentially enable more flexibility in sequence design,^41^ additional eddy‐current compensation^42^ and the removal of fold‐in artefacts but also enables exploring alternative encoding strategies.

The short echo‐spacing attainable with the presented gradient insert design allows substantial increases in spatiotemporal resolution of both single‐ and multiecho EPI fMRI experiments while also greatly reducing image distortions,^43^, ^44^ which was also observed in our in vivo example. The high gradient performance can also be used to explore high‐resolution, single‐shot, echo‐volume imaging with increased sensitivity offered by the short echo‐spacing.^1^ Additionally, general rapid sequences such as RARE and RAGE, but also (multiline) balanced steady‐state free precession and GRASE, can benefit from short readout gradients by expected increases in scan efficiency, shorter echo times, point spread function reductions and less image distortions.^45^, ^46^

In conclusion, the presented gradient insert design shows that high gradient performance (G = 200 mT/m and 1300 T/m/s) can be achieved with short installation times (15 min) and without large modifications to existing system hardware. In addition, the coil can offer a substantial increase in scan efficiency and spatiotemporal resolution for a wide range of (f)MRI applications, and importantly, its potential to study fine‐scale brain activity, but also the supporting structural architecture, at the mesoscopic scale.

## Supporting information


**Figure S1.** The exact winding positions of the gradient insert
**Figure S2**. A volunteer in the gradient insert with an fMRI mirror system for visual stimuli.
**Figure S3**. A schematic representation of gradient coil and relevant dimensions with the fMRI mirror system for visual stimuli.
**Figure S4**. In‐vivo example of geometrical distortion from the non‐linear gradient insert field before (middle) and after (right) geometry correction of the non‐linear gradient insert field. The data was acquired using a 3D‐GRE scout scan with 2.3 mm isotropic resolution and readout in the z‐direction (SI/FH). The same acquisition was repeated using the whole‐body gradients (left) to be used as a reference.
**Table S1.** The exact winding positions in meters with respect to the center of the conductors. The coil is symmetric around the center, so the complete winding configuration can be obtained by mirroring the given values.
**Table S2**. acoustic noise measurement for the EPI‐scans done in this paper shown with relevant sequence parameters. Acoustic noise was measured in terms of the peak sound level (LC_peak_), A‐weighted equivalent sound level for 1 s (LA_eq_) and the peak A‐weighted sound level recorded with fast time‐filtering.

## Data Availability

The data that support the findings of this study are available from the corresponding author upon reasonable request.
