# Supplementary material for: A plug‐and‐play, lightweight, single‐axis gradient insert design for increasing spatiotemporal resolution in echo planar imaging‐based brain imaging
Source: NMR Biomed. 2021 Feb 22;34(6):e4499. doi: 10.1002/nbm.4499 (PMC8244051; doi:10.1002/nbm.4499)
Supplement: Supplementary file 1 — Figure S1. The exact winding positions of the gradient insert Figure S2. A volunteer in the gradient insert with an fMRI mirror system for visual stimuli. Figure S3. A schematic representation of gradient coil and relevant dimensions with the fMRI mirror system for visual stimuli. Figure S4. In‐vivo example of geometrical distortion from the non‐linear gradient insert field before (middle) and after (right) geometry correction of the non‐linear gradient insert field. The data was acquired using a 3D‐GRE scout scan with 2.3 mm isotropic resolution and readout in the z‐direction (SI/FH). The same acquisition was repeated using the whole‐body gradients (left) to be used as a reference. Table S1. The exact winding positions in meters with respect to the center of the conductors. The coil is symmetric around the center, so the complete winding configuration can be obtained by mirroring the given values. Table S2. acoustic noise measurement for the EPI‐scans done in this paper shown with relevant sequence parameters. Acoustic noise was measured in terms of the peak sound level (LCpeak), A‐weighted equivalent sound level for 1 s (LAeq) and the peak A‐weighted sound level recorded with fast time‐filtering. [file NBM-34-e4499-s001.docx]

**Supporting information captions**

**Figure S1:**


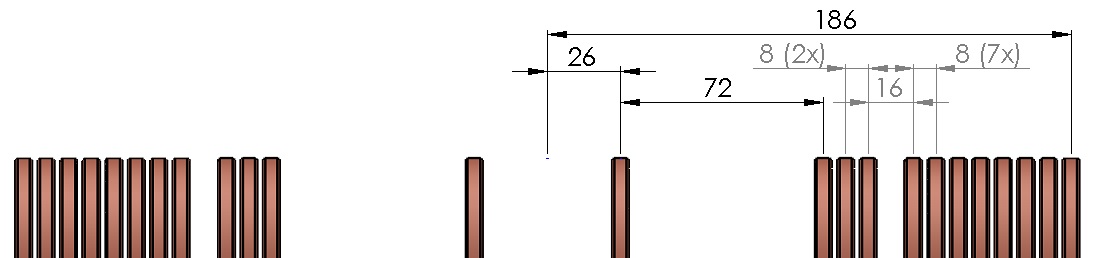


Figure S1 The exact winding positions of the gradient insert

**Table S1:**

**Table S1** The exact winding positions in meters with respect to the center of the conductors. The coil is symmetric around the center, so the complete winding configuration can be obtained by mirroring the given values.

| **Winding positions in meters** | | | | | | | | | | | |
| --- | --- | --- | --- | --- | --- | --- | --- | --- | --- | --- | --- |
| 0.026 | 0.098 | 0.106 | 0.114 | 0.13 | 0.138 | 0.146 | 0.154 | 0.162 | 0.17 | 0.178 | 0.186 |

**Figure S2:**

**
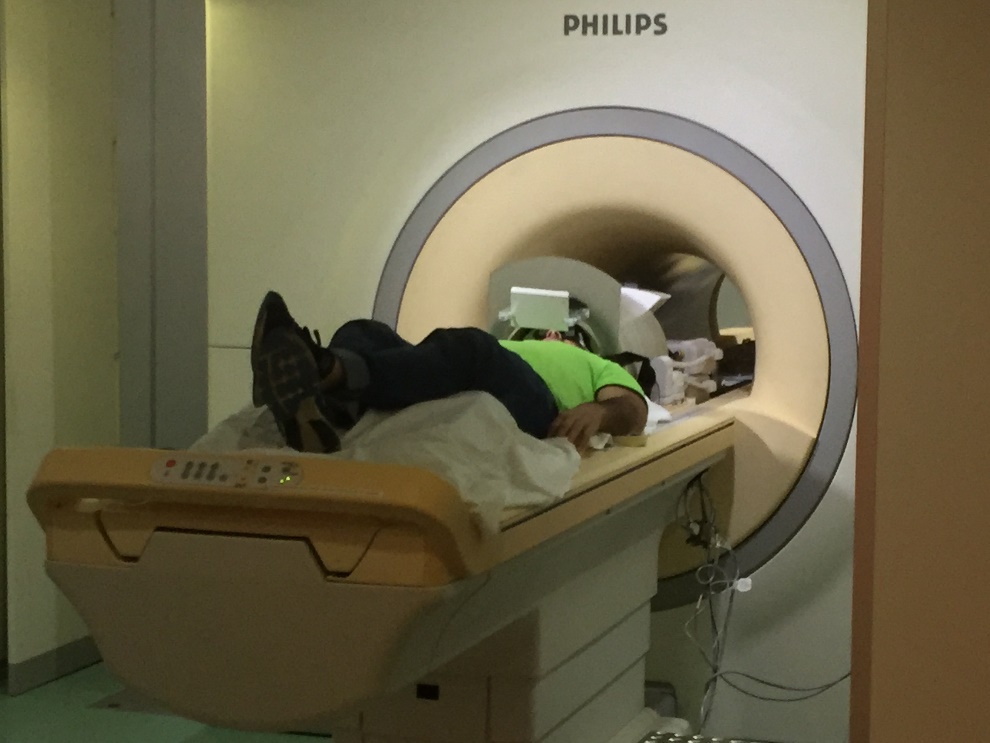
**

Figure S2 A volunteer in the gradient insert with an fMRI mirror system for visual stimuli.

**Figure S3:**

**
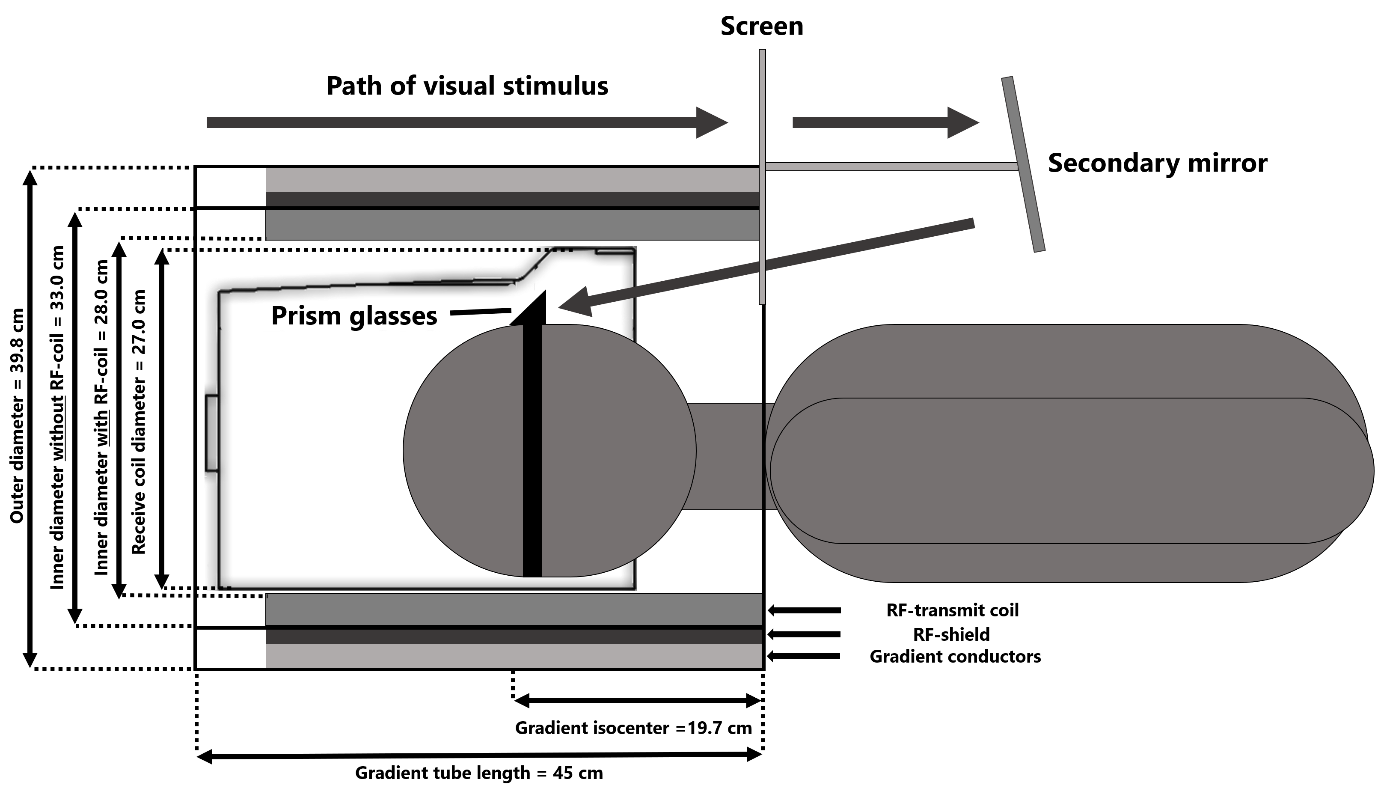
**

Figure S3 A schematic representation of gradient coil and relevant dimensions with the fMRI mirror system for visual stimuli.

**Figure S4**

**
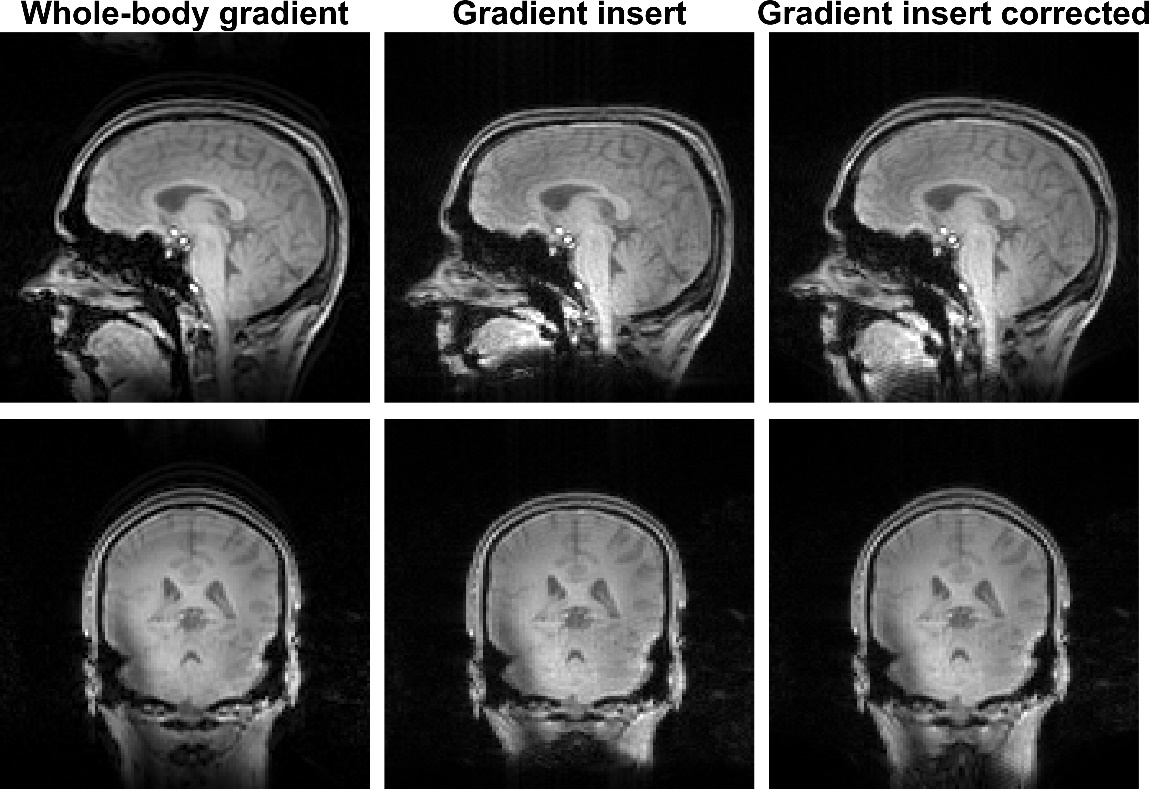
**

Figure S4: In-vivo example of geometrical distortion from the non-linear gradient insert field before (middle) and after (right) geometry correction of the non-linear gradient insert field. The data was acquired using a 3D-GRE scout scan with 2.3 mm isotropic resolution and readout in the z-direction (SI/FH). The same acquisition was repeated using the whole-body gradients (left) to be used as a reference.

**Table S2**

**Table S2**: acoustic noise measurement for the EPI-scans done in this paper shown with relevant sequence parameters. Acoustic noise was measured in terms of the peak sound level (LC­_peak_), A-weighted equivalent sound level for 1 s (LA_eq_) and the peak A-weighted sound level recorded with fast time-filtering.

| **EPI scan** | **Readout duration (ms)** | **Echo-spacing (µs)** | **G_max_ (mT/m)** | **Slew rate (T/m/s)** | **LC_peak_ (dB)** | **LA_eq_ (dBA)** | **Peak sound level (dBA)** |
| --- | --- | --- | --- | --- | --- | --- | --- |
| **2D EPI  (whole-body, figure 6a)** | 152.3 | 797 | 40 | 200 | 123.9 | 112.4 | 119.0 |
| **2D EPI  (insert, figure 6b)** | 152.3 | 797 | 40 | 200 | 119.2 | 106.6 | 109.2 |
| **2D EPI  (insert, figure 7b)** | 40.3 | 424 | 85 | 616 | 121.4 | 106.4 | 111.7 |
| **2D EPI  (insert, figure 6c)** | 73.9 | 387 | 96 | 800 | 121.6 | 106.7 | 111.0 |
| **2D EPI  (insert, figure 6d)** | 65.6 | 344 | 112 | 1290 | 124.8 | 111.6 | 116.7 |
